# Orientation detection for self-calibrating neuromorphic systems

**DOI:** 10.3389/fncom.2026.1878557

**Published:** 2026-10-23

**Authors:** Alejandro Pequeño-Zurro, Chiara Bartolozzi, Elisabetta Chicca

**Affiliations:** 1 Bio-Inspired Circuits and Systems (BICS) Lab, Zernike Institute for Advanced Materials, University of Groningen, Groningen, Netherlands; 2 Groningen Cognitive Systems and Materials Center (CogniGron), University of Groningen, Groningen, Netherlands; 3 Event-Driven Perception for Robotics, Istituto Italiano di Tecnologia (IIT), Genova, Italy

**Keywords:** continual learning, energy efficiency, event-driven processing, neuromorphic touch, receptive fields (RFs), spiking neural networks (SNNs), unsupervised learning

## Abstract

Bioinspired sensory architectures help build adaptive touch systems with efficient hardware implementations. Orientation detection is a fundamental capability for manipulating objects in real-world scenarios, with diverse applications in robotic manipulation and manufacturing assessment processes. In this work, we built spiking neural networks that combine biologically inspired receptive fields with unsupervised, local, spike-driven learning rules and self-regulating network dynamics to achieve orientation detection. The system is fully compatible with current neuromorphic hardware that operates in online learning conditions. Receptive fields were constructed using predefined connectivity based on biological findings in the somatosensory system, and the system was evaluated on edge orientation discrimination tasks against standard approaches in artificial systems. The system produces sparse, compressed neural representations, supporting energy-efficient neuromorphic implementations. Results show that random and unstructured receptive fields, which spread their inputs across the skin, outperform the regular kernel receptive field on this spatial discrimination task. The advantage is largest at fine angular resolution and small stimulus offset. These findings demonstrate that receptive field design is a key determinant of performance and present a biologically inspired framework for building self-calibrating, low-power tactile perception systems.

## 1 Introduction

Touch is a fundamental sensory modality for biological systems. Tactile representations of the physical world help navigate unstructured environments and provide extremely accurate dexterity in manipulation. To bridge the gap between artificial systems and the real world, we need to equip them with the same capabilities seen in nature (Garcia et al., 2009; Dahiya et al., 2009). Although several improvements in electronics and sensors have enabled miniaturization and increased the number of tactile sensors in the system, we remain constrained by architectural limitations. Traditional frame-based sensing relies on sampling at a fixed rate for highly dynamic phenomena; this results in data that are redundant when sensory signals are slowly changing, but might not adequately capture the evolution of extremely fast signals. This problem increases as the number of sensors scales up to match those of biological systems (Kim et al., 2014; Sundaram et al., 2019). Additionally, the sensing readout of traditional systems exhibits latencies of 100 ms, which are not compatible with fast sensorimotor control loops involved in dexterous object manipulation, for example, when counteracting slip, dynamic regrasping, or following objects' contours.

Neuromorphic, Event-driven sensing technologies (Lichtsteiner et al., 2008) are a perfect fit for addressing these challenges, given their asynchronous and adaptive sampling strategy, which can also be coupled with neuromorphic spike-based processing hardware that mimics the efficiency of the biological somatosensory system (Oddo et al., 2011; Tee et al., 2015). In this new paradigm, the sensors output discrete events rather than full taxel frames, adapting the sampling rate to the dynamics of the tactile signal. The significant decrease in the amount of generated data enables the system to achieve ultra-low latency and power consumption, especially in the absence of contact (Funk et al., 2024). If endowed with online learning capabilities, the neuromorphic spiking neural networks coupled with neuromorphic sensors can produce efficient, edge-processing systems that build representations of the real world, as biological systems do (Khacef et al., 2023), improving the system's computational efficiency and robustness (Khacef et al., 2023; Yi et al., 2023) and paving the way toward decentralized sensing intelligence (Li et al., 2021) with computational capabilities at the edge. This will bring improvements in autonomous robotics, prosthetic devices, and manipulation technologies (Liu et al., 2022; Tong et al., 2024; Zhu et al., 2025; Kudithipudi et al., 2025; M Ferreira et al., 2025).

Toward the design of a neuromorphic chip capable of edge processing of tactile stimuli, we draw inspiration from specific properties of biological systems and computational neuroscience models of learning. From the peripheral nervous system, mechanoreceptors convert skin deformations into spike trains that travel all the way to the somatosensory cortex, through the spinal cord, medulla, and thalamus (Delhaye et al., 2018). Several authors have studied the different types of mechanoreceptors that elicit tactile perception in primates. This work draws motivation from the work of Pruszynski and Johansson (2014), which shows that orientation selectivity emerges in first-order neurons in the peripheral nervous system. In other words, afferent nerves that combine the input from mechanoreceptors in the glabrous skin show orientation selectivity. The output activity of these neurons is correlated to the orientation of the tactile stimuli, indicating that this feature is already represented at the peripheral level rather than exclusively being processed in higher cortical areas (DiCarlo et al., 1998). Studies of the structure of their receptive fields reveal a distinctive pattern that can help unravel key findings about their computational properties. First, their shape is defined by clusters of high sensitivity regions, separated into patchy zones across the skin (Wessberg et al., 2003; Pruszynski and Johansson, 2014). Second, they present heterogeneous size with smaller receptive fields covering fingertips, and wider fields covering areas in the palm and other sparsely populated skin areas (Johansson and Åke B. Vallbo, 1979; Johansson and Vallbo, 1983). Third, the receptive fields of all neurons overlap, the clusters are distributed, and their sparse activation contributes to efficient tactile encoding (Johansson, 1976; Hsiao et al., 2009). Finally, they combine their outputs in the second-stage somatosensory cortex, resulting in a nonlinear integration of features that leads to spatial invariance (Thakur et al., 2006). Our model proposed in this work includes the shape and the overlap properties of the biological receptive field previously mentioned.

The emergence of the computational properties of networks of neurons in biology relies on local information that is used to modify the strength of the synaptic connections between neurons that exchange information. These mechanisms have been modeled within a theoretical framework for synaptic memory consolidation, emphasizing multistable synapses in spike-based driven learning for long-term retention (Fusi, 2002, 2003). Brader et al. (2007) developed this framework by proposing a calcium-based synaptic plasticity model to learn input representations using internal variables that bridge biological plasticity mechanisms with neuromorphic-compatible supervised systems. Diehl and Cook (2015) present an implementation of an unsupervised learning spike timing dependent plasticity rule (STDP) that self-organizes input representations. This method leverages the network dynamics of lateral inhibition to balance output representations without any supervision.

In this work, we investigated how to leverage these insights and current advancements in neuromorphic chip design to develop robust, self-calibrating, and efficient tactile edge processing, offering solutions to unsolved sensing challenges. Early examples of this approach exhibited robustness and adaptation in object manipulation and shape detection in robotic systems (Rongala et al., 2017; Martinez-Hernandez et al., 2020; Brayshaw et al., 2024; Xu et al., 2025; Lu et al., 2026), and prosthetics and autonomous control (Kumar et al., 2022; Rahiminejad et al., 2021). Overall, tactile neuromorphic systems provide the key ingredients for building real-time, low-energy, adaptive solutions for hardware perception control systems (Macdonald et al., 2022; Xu et al., 2025).

We employed bioplausible spike-based learning rules (Brader et al., 2007) that can be integrated within spiking neural networks and deployed on mixed-mode subthreshold neuromorphic hardware (Greatorex et al., 2025) and offer significant energy efficiency advantages over standard hardware approaches (Gautam and Kohno, 2021; Ostrau et al., 2022; Gebregiorgis et al., 2025). We instantiated the local learning rules in an unsupervised learning context that aligns with the natural paradigm of learning by exploration, allowing the network dynamics to negotiate the input representations without supervision. Unlike previous works (Dabbous et al., 2021), we tested the preprocessing capabilities of the receptive fields observed in the touch sensory modality using unsupervised learning rules that reveal the capabilities of the primary stages of the somatosensory system. Compared to Pruszynski's statistical framework (Pruszynski et al., 2018), this work presents a mechanistic, biologically grounded model that captures tactile edge orientation through unsupervised local learning, demonstrating how energy-efficient neuromorphic event-based systems can be built.

## 2 Methods

### 2.1 Spiking neural network for the emergence of orientation tuning

Figure 1 shows the overall system leading to tactile orientation classification. It is composed of a sensing layer, i.e., a skin patch, an encoding layer that transforms the analog activation of the tactile transducers into spike trains, and a two layer spiking neural network. The first layer of neurons had fixed receptive fields (synaptic weights) that integrated the activity of multiple tactile sensing elements (taxels) across the skin patch. The connections between the first and second layers were instead learnt through unsupervised spike-driven synaptic plasticity, leading to orientation tuning in the output layer. The skin patch was simulated as a tactile surface inspired by the iCub humanoid robot capacitive skin (Bartolozzi et al., 2016). The skin patch is composed of a matrix of 15 × 15 tactile elements (taxels). Each taxel outputted a floating point number that corresponds to the pressure due to the contact of a stimulus pressed on top of the skin. The dataset was generated by simulating the pressure from a bar (width of two taxels, length of 15 taxels) at different orientations, distributed evenly around 360 degrees. The dataset variability accounts for the offset in the spatial dimensions of the surface (x, y) and the variability of the taxel activation, where the bar is partially occluded due to the angle of incidence, simulating partial pressure of the bar. Every sample was then represented by a 15-by-15 matrix, with values between 0 and 1 corresponding to the voltage range of the taxel sensor. In this study, we assumed an instantaneous stimulus application, discarding the temporal dynamics of the onset of the touch.

**Figure 1:**
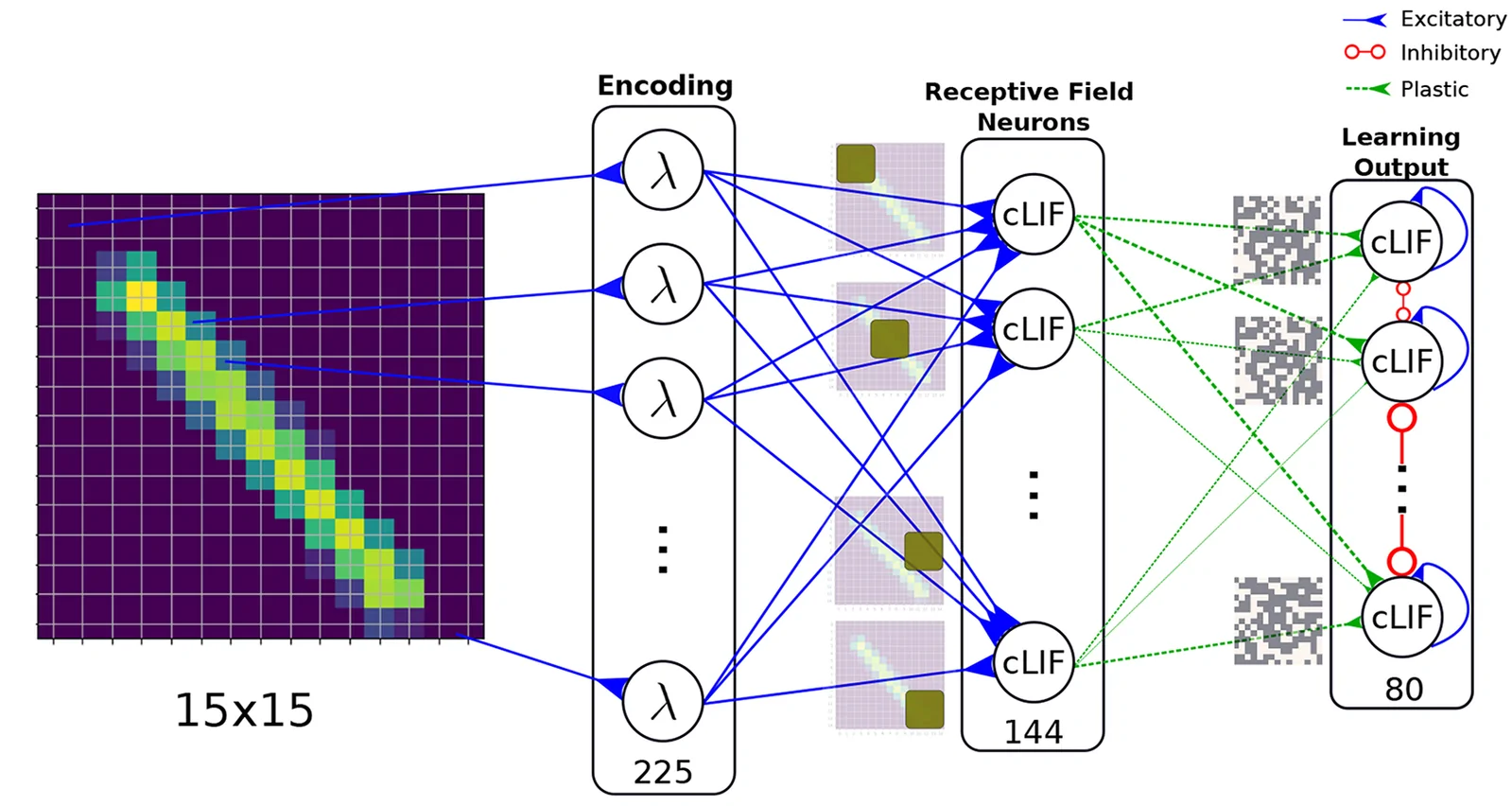
Classification system. The system comprises a receptive surface composed of tactile-based sensors arranged in an array, an encoding layer that encodes analog sensor data into spike events, and a two-layer spiking neural network. The neural network is built as a layer of Leaky-Integrate-and-Fire neurons whose synapse matrix represents a generated receptive field according to the conditions explored in the manuscript, and a second population layer of Leaky integrate-and-fire neurons with plastic synapses following an unsupervised calcium-based rule. The system architecture learns relevant features for discriminating orientations in the output population.

The encoding layer generated Poisson spike trains with a frequency determined by the taxel's activation (i.e., the instantaneous input pressure), similarly to the slow adaptive (SA) type of mechanoreceptors, and each spike was randomly generated from a Bernoulli distribution. The fixed receptive fields of the first neuron layer were modeled following the random interleaved active sensing fields of first order afferents (Pruszynski and Johansson, 2014) and compared with conventional artificial configurations described in Section 2.3. The weighted input from the receptive field was integrated with a CuBa-LIF neural model as described in Section 2.2. The first layer comprised 144 neurons. The output layer contained 80 CuBa-LIF neurons, each neuron received input from all neurons in the first layer through plastic synapses, along with self-recurrent excitation and lateral inhibition. The synapses remained plastic during the network training phase (80% of the total dataset), and their weights remained frozen during the evaluation phase. The performance was measured in terms of classification accuracy and network sparsity. In the evaluation phase, we stimulated the skin patch with bars representing the orientation classes. The output neurons had previously learnt representation features from the receptive field layer in an unsupervised learning process. Every output neuron followed a unique tuning curve that represents the input space. We labeled each neuron based on the input orientation that corresponds to its maximum spiking activity. The neurons in the output were then grouped by class; the decision for the input class was inferred from the maximum spiking activity per class. The spiking activity per class was defined as the average population activity for each class in the network previously labeled.

The network consisted of 225 input encoding neurons, followed by the receptive field layer of 144 neurons, and an output layer of 80 neurons. The encoding layer was defined by mapping one to one the taxels in the surface. The number of receptive field neurons had been empirically chosen to minimize the randomicity effect of the generation of receptive fields. The output layer size had been selected to ensure representation of all classes in the population vote after the labeling phase. While the network is easily scalable, we chose a compact network compatible with current ultra-low-power neuromorphic hardware architectures, including mixed analog-digital signal chips such as BrainScaleS-2, Dynap-se2, or Texel (Pehle et al., 2022; Richter et al., 2024; Greatorex et al., 2025). The experimental design, dataset construction and parameter optimisation search for every tuned parameter and condition are given in Supplementary material S1, S5, S7.

### 2.2 Neuron model: Current-Based Leaky Integrate-and-Fire (CuBa-LIF)

The CuBa-LIF neuron model (Equations 1–4) was adopted from Cramer et al. (2020) and Zenke and Vogels (2021). We chose the CuBa-LIF model, which includes synaptic current dynamics, over the idealized LIF neuron counterpart, while remaining compatible with current neuromorphic hardware without the increased complexity of models such as the adaptive exponential integrate-and-fire (AdEx) neuron model (Brette and Gerstner, 2005). The membrane potential $U_{i}^{\left(l\right)}$ of neuron *i* in layer *l* evolves as


$$\begin{array}{l} \tau_{mem}\frac{dU_{i}^{\left(l\right)}}{dt}=-\left(U_{i}^{\left(l\right)}-U_{rest}\right)+RI_{i}^{\left(l\right)}, \end{array}$$ (1)

where τ_*mem*_ is the membrane time constant, *U*_*rest*_ the resting potential, *R* the input resistance (set to 1 for simplification), and $I_{i}^{\left(l\right)}$ the synaptic input current. The synaptic current dynamics are


$$\begin{array}{l} \tau_{syn}\frac{dI_{i}^{\left(l\right)}}{dt}=-I_{i}^{\left(l\right)}\left(t\right)+\underset{j}{\sum }W_{ij}S_{j}^{\left(l-1\right)}\left(t\right)+\underset{j}{\sum }V_{ij}S_{j}^{\left(l\right)}\left(t\right), \end{array}$$ (2)

with τ_*syn*_ the synaptic time constant, *W*_*ij*_ feedforward weights, and *V*_*ij*_ recurrent and lateral inhibition weights used in the output layer.

The discrete-time implementation becomes


$$\begin{array}{l} I_{i}^{\left(l\right)}\left(t\right)=\alpha I_{i}^{\left(l\right)}\left(t-1\right)+\underset{j}{\sum }W_{ij}S_{j}^{\left(l-1\right)}\left(t\right), \end{array}$$ (3)

$$\begin{array}{l} U_{i}^{\left(l\right)}\left(t\right)=(\beta U_{i}^{\left(l\right)}\left(t-1\right)+I_{i}^{\left(l\right)}\left(t\right))\left(1-U_{reset}\right), \end{array}$$ (4)

where $\alpha =e^{-dt/\tau_{syn}}$ and $\beta =e^{-dt/\tau_{mem}}$ are decay factors. We defined *U*_*reset*_ as the post-spike reset of the membrane potential.

### 2.3 Spatial receptive fields

A receptive field is the specific sensory input a neuron or computational unit processes to produce its response. In biological systems, individual mechanoreceptors spatially distributed in the skin are combined at the peripheral nervous system and at central relays in the spinal cord and thalamus before arriving at the primary somatosensory areas. The receptive fields of first order afferents that show orientation selectivity in the human hand can be modeled as unstructured clusters of mechanoreceptors distributed randomly across skin patches (Wessberg et al., 2003; Pruszynski and Johansson, 2014). In our artificial system, we randomly distributed taxel inputs to cover the entire surface area and assigned clusters of three adjacent taxels, as shown in the pseudocode of Listing 1. To evaluate the advantages of this type of receptive field, which we denominated in the text as *unstructured*, we compared system performance against two alternative receptive field generation methods: *randomly* distributed and *kernel*-based surface coverage. The *kernel* fields were implemented as square local connectivity windows that tile the input surface with overlap, representing the standard localized sampling strategy. In every receptive field condition, every weight in the receptive field matrix was assigned a binary weight (0 = not activated, 1 = activated) representing the surface input space with 16 activated weights per neuron (Supplementary material S6).

Listing 1 Unstructured Receptive Field generation


#Generating unstructured cluster
G = all input pixels
guaranteed = shuffled seeds (>=1 per neuron)
random_pool = shuffled(G * overlap)
W = zeros(inputs, neurons)
for n in neurons:
    for s in range(fields):
        seed = pop(guaranteed) if s==0
                 else pop(random_pool)
        activate(seed, n)
        k = 1
        for nb in neighbors(seed):
            if k == size_k: break
            if nb in random_pool and
                activate(nb, n):
                remove(nb, random_pool)
                k += 1
    add_spare_random_pixels(n)
return W

Figure 2 shows examples of the receptive fields across all methods compared in this work. In the left column, a single receptive field (in gray) of a neuron is superimposed on the skin patch with a visualization of the activation of the taxels (in red) due to a bar pressed on the skin patch. The second column shows all the receptive fields in the middle layer of Figure 1. The right column shows the total coverage of the input space by overlapping all receptive fields. The total number of active weights in the full layer was the same for all the receptive field conditions since every neuron presented the same size of the receptive field. However, randomization and spatial constraints within each receptive field condition modified the total spatial coverage at the layer level. The *kernel* condition was fully spatially constrained with no randomization. The *unstruct* condition uses randomization to select the initial points of the clusters, but it imposes a spatial constraint that connects contiguous taxels within small clusters. The generation with the *random* condition uses taxel randomization and imposes no spatial constraints. The unstructured method, inspired by the biological findings, features multiple clusters of randomly distributed weights; it seems to localize local features while randomly covering the input space. The generation algorithm is defined in Listing 1 where fields=5 is the number of sub-clusters per unstructured RF neuron, size_k=3 the taxel number per sub-cluster, overlap=12 the number of copies of the input pool available for sampling, spare=1 the extra random taxels per neuron, neighbors the eight grid offsets used to grow each sub-cluster into its surroundings, and samples=16 the taxels per random RF neuron. The output projection (144 → 80) is all-to-all plastic, and lateral inhibition and self-excitation are 80 × 80 (off-diagonal inhibitory, identity excitatory).

**Figure 2:**
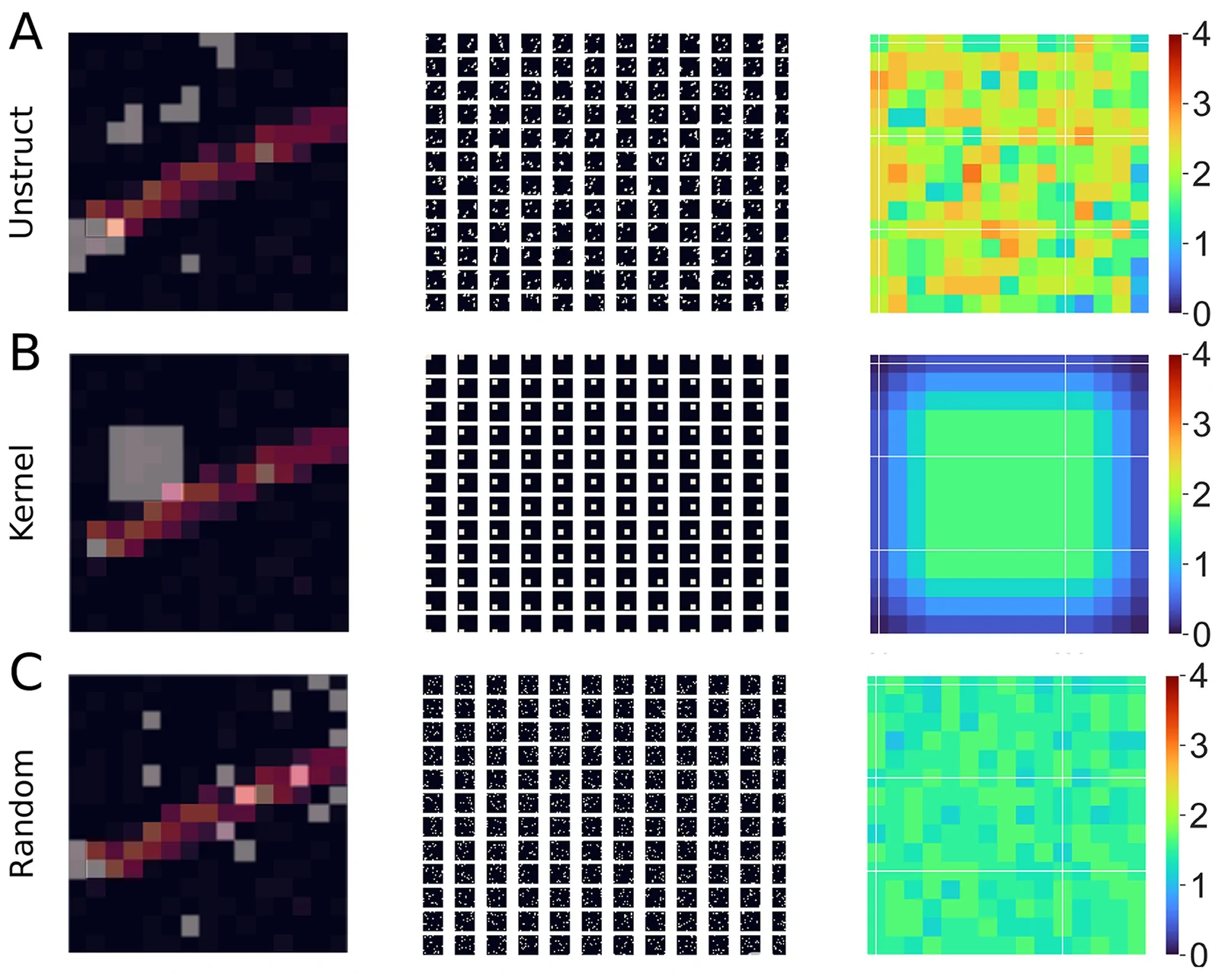
Receptive fields and input space coverage. Representation of the three methods for the generation of receptive fields tested in this manuscript. Row **A**, the unstructured kernel was generated by randomly distributing taxel clusters in the input space. Row **B**, squared kernels were generated to cover the input space evenly. Row **C**, single elements were randomly distributed to cover the input space. For all conditions, we imposed full coverage of the input space during receptive field generation. Columns described the input space with a sample receptive field for each kind, all receptive fields in the middle layer, and full coverage of the input space overlapping all neural fields. Examining the coverage differences reveals how the middle layer samples the input space.

### 2.4 Online local learning: spike-driven synaptic plasticity

The weights of the connections between the first layer neurons and the output layer were modified following a biologically plausible learning rule, that relies only on local information, is capable of supporting long-term memory, and is suitable for hardware implementation (Brader et al., 2007). This learning rule utilizes spike-based learning dynamics: the synapse gets potentiated or depressed based on the spiking activity of the postsynaptic and the presynaptic neurons. The most relevant example of this type of learning rule is STDP (Spike Timing Dependent Plasticity) (Caporale and Dan, 2008). Instead of monitoring the precise timing of the pre- and post- synaptic spikes as in STDP, the model relies on a Calcium trace that correlates with the activity state of the postsynaptic neuron. The Calcium trace dynamics follow Equation 5:


$$\begin{array}{l} \frac{dC\left(t\right)}{dt}=-\frac{1}{\tau_{C}}C\left(t\right)+J_{C}\underset{i}{\sum }\delta \left(t-t_{i}\right) \end{array}$$ (5)

where δ(*t*−*t*_*i*_) is the spike train of the postsynaptic neuron, *J*_*C*_ defines the efficacy or the amount of change in the trace per spike, while $\frac{1}{\tau_{C}}$ is the exponential decay of the trace defined by its time constant τ_*C*_.

The synaptic weight modification was driven by the presynaptic spikes as described in Equation 6. At the time of the pre-synaptic spike *t*_*pre*_, the synaptic weight is potentiated or depressed based on the value of the membrane potential of the postsynaptic neuron (*V*) (above or below a threshold (θ_*V*_), respectively), and the state of its Calcium trace: when the trace is within the potentiation range thresholds ($\theta_{\text{pot}}^{l},\theta_{\text{pot}}^{h}$) or depression range thresholds ($\theta_{\text{dep}}^{l},\theta_{\text{dep}}^{h}$). The condition of the membrane potential adds stochasticity to the learning rule.


$$\begin{array}{l} X\to \{\begin{array}{cc} X+a & \text{if }V\left(t_{\text{pre}}\right)>\theta_{V}\text{ and }\theta_{\text{pot}}^{l}<C\left(t_{\text{pre}}\right)<\theta_{\text{pot}}^{h}\\ X-b & \text{if }V\left(t_{\text{pre}}\right)\leq \theta_{V}\text{ and }\theta_{\text{dep}}^{l}<C\left(t_{\text{pre}}\right)<\theta_{\text{dep}}^{h} \end{array} \end{array}$$ (6)

Each synapse is binary and bistable. This means that it only has two possible values (active, or non-active). *X* is an internal trace that gets potentiated or depressed at the occurrence of a presynaptic spike following Equation 6, and decays over time toward one of the two bistable states following Equation 7 with time constants α and β, respectively. Each synapse will return to its stability point unless it gets potentiated or depressed consecutively until crossing the threshold θ_*x*_.


$$\begin{array}{l} \frac{dX}{dt}=\{\begin{array}{cc} \alpha & \text{if }X>\theta_{X}\\ -\beta & \text{if }X\leq \theta_{X} \end{array} \end{array}$$ (7)

In the original model, synaptic plasticity relies on an external supervisory signal, denoted as the *teacher signal*, whose role is to bias the postsynaptic activity of the neuron selected to encode a particular input class. The teacher signal explicitly assigns every output neuron to a specific class pattern. However, following examples applied to other learning rules (Diehl and Cook, 2015; Khacef et al., 2020), we proposed that the spike-based learning rule described above can learn relevant features of the whole dataset in an unsupervised fashion if there is negotiation and competition at the network architecture level. To achieve this, we combined self-excitation and lateral inhibition across the output learning layer (Figure 1). Self-excitation acted as an internally generated teaching signal. It increased the neuron firing rate, therefore generating elevation in the calcium trace, bringing the neuron into a state which supports synaptic potentiation depending on the input spike trains. Lateral inhibition made different neurons selective to different patterns of activation, as an increase in spiking activity of a neuron silences the rest of the population. Lateral inhibition therefore increased the network's capacity to learn multiple activation patterns. The combination of these two types of connectivity generated a self-regulatory behavior within the neural population.

For this model to work as intended, learning relevant features from the dataset while avoiding catastrophic forgetting, it required tuning of the parameters of the network which involved the parameters of the spike-driven synaptic learning rule, but also the weights of the network, all listed in Table 1. Hyperparameters were optimized using Optuna, an automatic hyperparameter optimisation framework based on Bayesian optimization with Tree-structured Parzen Estimator (TPE) sampling (Akiba et al., 2019). To guarantee a fair comparison of performance we ran the hyperparameter optimisation for every condition of the receptive field generation.

**Table 1:** Parameters of the system, calcium-based synaptic plasticity rule, and recurrent network dynamics.

Receptive Field Layer	
***w*_*exc*_** τ_*EPSP*_	Fixed excitatory projection weight from sensory inputs to receptive field neurons. Time constant of excitatory postsynaptic currents in the receptive field layer.
**Spike driven, Calcium Based Synaptic Plasticity**	
τ_*w*_ **τ_*c*_**	Time constant governing slow bistable drift of synaptic efficacy. Calcium trace decay constant controlling temporal integration of activity.
*a*	Rate of potentiation update during event-driven plasticity.
*b*	Rate of depression update during event-driven plasticity.
α	Strength of drift toward the potentiated synaptic state.
β	Strength of drift toward the depressed synaptic state.
θ_*w*_	Threshold separating weak and strong stable synaptic states.
θ_*v*_	Postsynaptic voltage threshold determining potentiation vs. depression.
Recurrent Output Layer Dynamics	
*J*_*eff*_	Effective synaptic scaling factor of plastic feedforward inputs.
*w*_*inh*_	Lateral inhibitory synaptic weight between output neurons regulating competition.
τ_*EPSP*_	Time constant of feedforward excitatory postsynaptic currents.
τ_*IPSP*_	Time constant of inhibitory postsynaptic currents.
*w*_*exc*_	Recurrent self-excitatory synaptic weight.
τ_*exc*_	Time constant of recurrent excitatory currents.
**θ_*pot, low*_**	Lower calcium threshold defining onset of potentiation window.
**θ_*pot, high*_**	Upper calcium threshold bounding potentiation window.
**θ_*dep, low*_**	Lower calcium threshold for depression window.
**θ_*dep, high*_**	Upper calcium threshold for depression window.
***c*_*eff*_**	Effective calcium coupling factor influencing synaptic updates.

Bold parameters indicate key parameters to tune with the optimizer per network condition.

### 2.5 Neuron labeling and population vote

Our system was trained using an unsupervised learning process, which better reflects how biological sensory systems acquire representations through unlabeled interaction with the environment. This paradigm also matches real-world scenarios, where users interact with the system naturally and the system must progressively learn relevant representations from experience without explicit supervision. Unsupervised learning requires a labeling phase in which every neuron is assigned a class based on its maximum output (Diehl and Cook, 2015; Khacef et al., 2020). After presenting the training dataset, we assigned each neuron to its correct class based on its maximum response. After labeling all the neurons, we grouped them into populations based on their labeled class. Every neuron then became part of a population that represented that class.

In the testing phase, the activity of each population of neurons was averaged across the whole population. The input class was inferred by selecting the label of the population with the highest activity. Classification accuracy was defined as the proportion of test stimuli for which the decoded class matches the ground-truth. With this readout, the network was effectively forced to base its decisions on population codes. If all populations were silent or similarly active, no class clearly wins the vote, and the classifier, then, could fail to produce correct outputs on any trial. Essentially, if the synaptic plasticity rule does not learn meaningful, class-specific activity patterns, decoding performance will remain at chance or very low, indicating that the network has not acquired useful representations. Equation 8 for labeling, where *j* indexes the output neurons, *c* is a class (bar orientation), *X*_*c*_ is the set of training presentations of class *c*, *s*_*j*_(*x, t*) is the spike of output neuron *j* at time step *t* during stimulus *x*, *T* is the number of time steps per stimulus, so ${\bar{r}}_{c}\left(j\right)$ is neuron *j*'s spike count summed over time and averaged over all presentations of class *c*, and label(*j*) is the class assigned to neuron *j*.

Decoding is unsupervised and uses no external classifier. After training, with plastic synapses frozen, the training set is presented once and each output neuron's mean spike count per class is recorded. The neuron is labeled with the class of its highest mean. Neurons sharing a label form that class's population. At test time the response to a stimulus is the per-class average spike count over each population, and the predicted class is the population with the highest mean (argmax, first index on ties). A class that receives no neuron during labeling cannot be selected. See Equation 9 where *x* is the test stimulus, *N*_*c*_ = {*j*:label(*j*) = *c*} is the population of neurons labeled with class *c*, the inner sum $\overset{T}{\underset{t=1}{\sum }}s_{j}\left(x,t\right)$ is the spike count of neuron *j* over the stimulus, and ŷ(*x*) is the predicted class.


$$\begin{array}{l} \text{label}\left(j\right)=\arg\underset{c}{\max}{\bar{r}}_{c}\left(j\right),\;{\bar{r}}_{c}\left(j\right)=\frac{1}{\left|X_{c}\right|}\underset{x\in X_{c}}{\sum }\overset{T}{\underset{t=1}{\sum }}s_{j}\left(x,t\right) \end{array}$$ (8)

$$\begin{array}{l} ŷ\left(x\right)=\arg\underset{c}{\max}\frac{1}{\left|N_{c}\right|}\underset{j\in N_{c}}{\sum }\overset{T}{\underset{t=1}{\sum }}s_{j}\left(x,t\right) \end{array}$$ (9)

## 3 Results

To validate the unsupervised spike-driven learning used in this work and unravel the role of different receptive fields in the emergence of orientation tuning in tactile systems, we evaluated classification accuracy of the trained networks, the sparsity of their activation during inference, and the capability of the network to support incremental learning.

### 3.1 Network regulation

In the context of unsupervised learning, the random connections between the first layer and the output layer ensure that different input pattern combinations of receptive fields (Figure 3) generate individual patterns of activation in the output neurons. The first neurons that produce spikes in the output layer will reinforce their activity through self-excitation and silence the activity of the neurons that receive a weaker input drive. Through the consequent synaptic potentiation, those neurons became selective to the input class.

**Figure 3:**
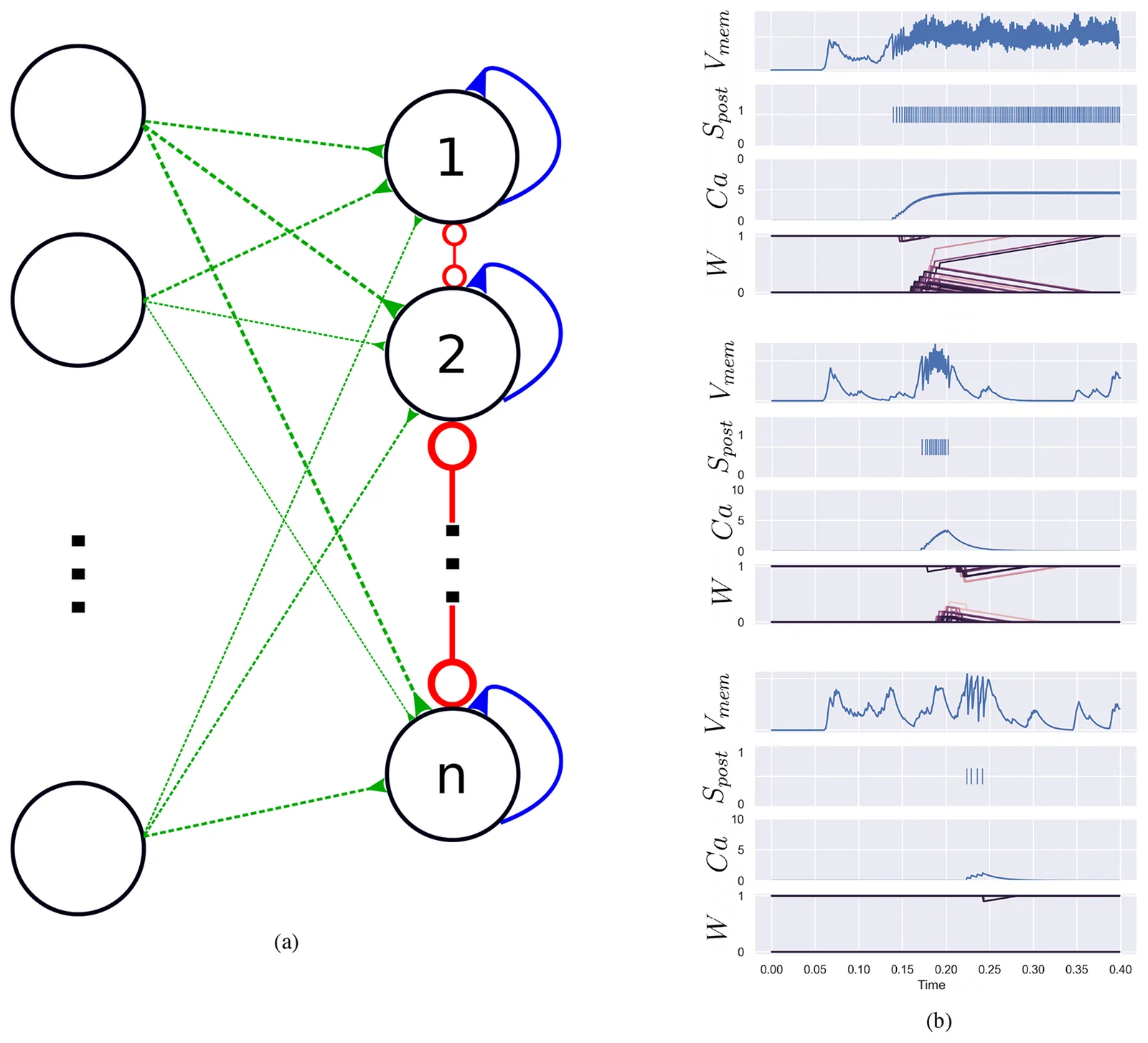
Self regulatory mechanisms supporting unsupervised spike-driven learning. **(a)** The connectivity pattern of the network features self-excitatory (blue), inhibitory (red), and plastic (green) synapses. **(b)** Traces of three neurons in the output layer over time after presenting one stimulus orientation. *S*_*post*_ is the postsynaptic evoked spikes, *V*_*mem*_ the membrane potential, *Ca* the calcium trace, and *W* the plastic weights of all of the input synapses for each of the three monitored neurons. The figure shows the regulatory mechanism that occurs in the dynamic interaction between plastic, self-excitatory, and lateral inhibitory synapses. In the example, only neuron 1 has its synapses potentiated in response to the presented pattern. Neuron 2 competes to learn the pattern but is inhibited by the activity of the reinforced neuron 1. Neuron n never becomes sufficiently polarized by the presented pattern (as seen in the calcium trace).

In Figure 3, we presented an example to demonstrate this self-regulatory mechanism of the network. We initially present a sample stimulus to the network's input. The neurons competed to learn a pattern not previously seen. Neuron 1 and Neuron 2 became active, and their calcium traces increased. Neuron n, with its randomly initialized synapses, did not become sufficiently active. Since Neuron 1 reached the calcium potentiation zone faster, the synapses whose inputs were getting presynaptic spikes got potentiated according to the stochastic learning rule (Section 2.4). Once those specific synapses were potentiated, the probability of increasing their spiking activity also increases. The lateral inhibition between the output of Neuron 1 to Neuron 2 and Neuron n decreased their spiking activity. Since these neurons were inhibited, their synapses did not reach the calcium trace threshold and were not ultimately potentiated. This is a regulatory property of the network that prevents all synapses to learn the same pattern of activation. The figure represents the temporal sequence of a single presentation. In the scope of the classification task we need to learn to classify different input patterns. In that case, we would present a different stimulus to the network, in that hypothetical case, Neuron 2 (or Neuron n) would get activated high enough to reach the potentiation range first, reinforcing some of its synapses. Which neuron gets active first, thereby driving the synapses toward potentiation or depression, is determined by their initial weight matrix. Since we randomly initialize the weight matrix for all output neurons, we ensured that every input pattern activates different output neurons, each strong enough to drive the plasticity rule. In the task to achieve this self-regulation across multiple activation patterns, the network's hyperparameters play a crucial role.

### 3.2 Performance accuracy

Once we found the key parameters to achieve self-regulatory behavior, we trained the network to classify the orientation of bars pressed on the skin patch and study the effect on classification accuracy of using different types of receptive fields (unstructured, kernel, random, described in Section 2.3 and represented in Figure 2). We tuned the hyperparameters of the network for each condition defined by the number of orientation classes, and the receptive field types to ensure maximum possible performance in all cases. Hyperparameter tuning procedure and parameters for each of the networks can be found in Supplementary material S7.

Every point in Figure 4 represents the mean and confidence interval of the testing accuracy for 15 random initializations of the network. Every seed the network is randomly initialized and the receptive fields are generated, moreover, the plastic synapses in the output layer are randomly initialized. The left side of Figure 4 shows the performance for increasing the number of classes (that corresponds to changing the minimum angle between possible stimulus orientations) with random receptive fields showing the best performance on average for all cases, and the unstructured receptive fields outperforming the kernel receptive field. For each receptive field condition, we compared the performance distributions pairwise and tested the differences for statistical significance. Significant differences are marked at the top of the figure, and the full statistical results are reported in Supplementary material S2. In all conditions, the classification accuracy decreases with the number of classes and the smaller discrimination angle. The right panel of Figure 4 illustrates the performance of the networks with variations in the dataset generation based on the offset of the bar location on the skin patch. Every unit of the offset represents a taxel on the tactile surface, representing the variability in which a neighbor taxel may be stimulated simultaneously in the x and y axes of the tactile surface. This procedure is further explained in Supplementary material S5. The variation of the offset has been measured in the figure at a constant minimum discrimination angle of 25.7° or 14 classes. As expected, the classification accuracy drops with the offset, below five taxels offset, the random and unstructured receptive fields show higher accuracy but more sensitivity with respect to the kernel condition. Above five taxels offset value, the performance decreases significantly for all conditions. The slope variation in each receptive field condition depends on the accuracy sensitivity to the network hyperparameters. Each point on the axis of Figure 4 has been tuned individually to maximize accuracy. Across conditions, the random receptive field also produced the lowest orientation error. Taking the 14-class task as a reference, its mean error was 1.05°, compared with 9.21° for the unstructured field and 20.67° for the kernel. Relative to the 25.7° spacing between classes (360°/14), the random field's error amounts to only 4% of a discrimination step, whereas the unstructured field and the kernel reach 36% and 80%, respectively. The full mean orientation errors are reported in Supplementary material S8. These results provide an estimate of the limits of tactile orientation detection for our system and highlight the spatial sensitivity of the construction methods for the different receptive fields.

**Figure 4:**
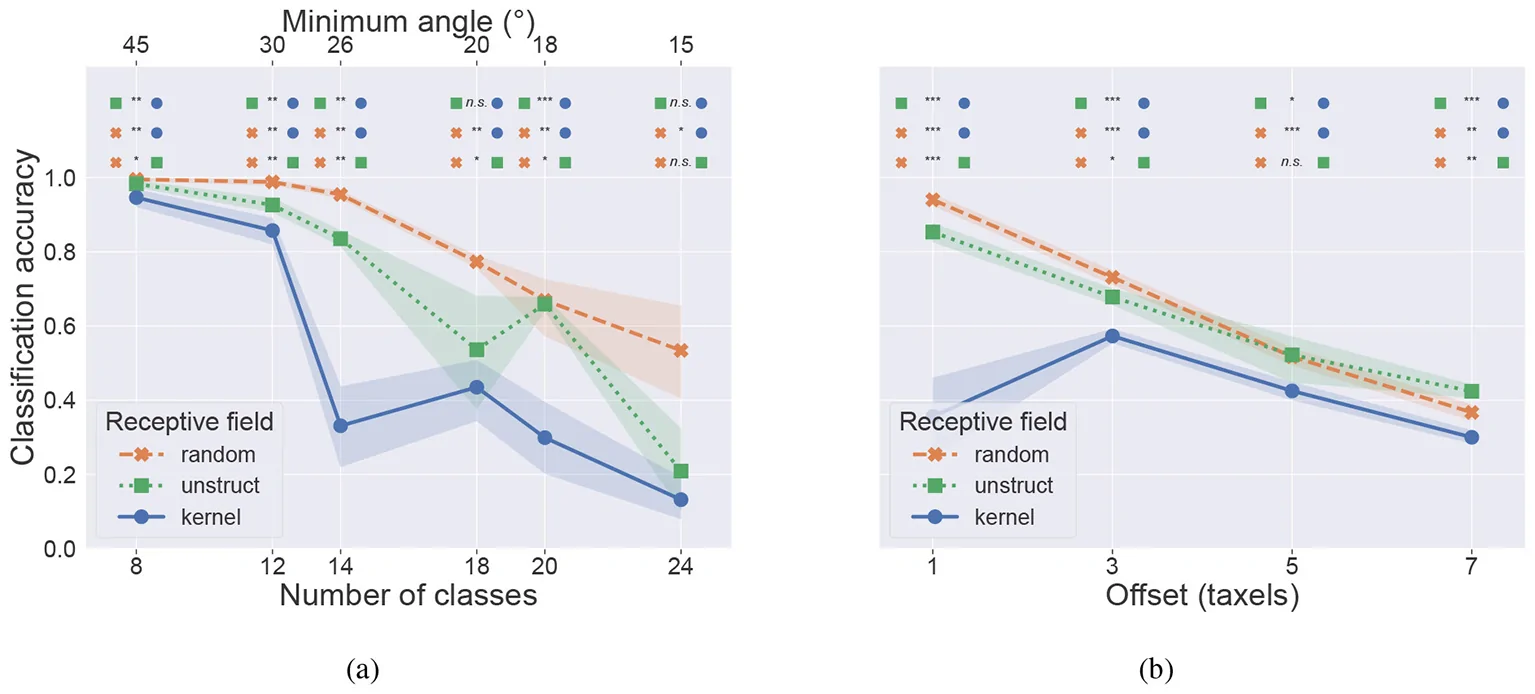
Comparative performance of the orientation classification network for different receptive fields. **(a)** classification accuracy decreases for increasing angle resolution between oriented bar presentations (corresponding to an increased number of classes). **(b)** classification accuracy decreases with an increased offset in the x and y displacement of the oriented bar on the skin patch. All values have been calculated over fifteen different random initializations; the hyperparameters of the networks have been previously optimized individually for each dataset and receptive field condition. The random and unstructured receptive fields outperform the kernel in most conditions, with slight superiority of the random condition. All the pair statistical significance tests shown on top of the figure have been calculated following the Wilcoxon signed-rank test, paired across seeds with a total of fifteen seeds per condition. Star convention on the plot is the conventional ^*^p < 0.05, ^**^p < 0.01, ^***^p < 0.001, n.s. for not significant.

### 3.3 Sparsity

Sparsity is a desirable property since it promotes efficient computation and class representation. Figure 5 shows the activity of the neurons in the two layers of the network during inference after training, with networks optimized to discriminate bar orientations separated by 25.7° between consecutive classes (or 14 classes). The activity of each neuron has been normalized for comparative purposes in spikes per second per orientation class. Every circle in the figure represents the activity of one neuron. The colors represent the orientation class, and the intensity represents the activation of each neuron individually. A non-active neuron with 0 spikes per second is represented with a transparent color, while the maximum neuron activation is represented with the maximum opacity. The figure represents comparatively visualization of the activation of the network. To measure quantitatively the activation of the network, we present Table 2. We analyzed network activity across fifteen random initializations for the 25.7° angle discrimination (14 classes). Every metric in the table points to a high degree of compression from the receptive field layer to the output layer, and the random condition shows the lowest degree of compression between the layers. The Gini coefficient and the Treves–Rolls sparseness *S* confirm that the output activation is highly sparse on average per stimulus even though the network is not specifically optimized for it. The random receptive field reaches the highest accuracy while remaining the least sparse at the output, which indicates a regulation of activity that retains the units needed for discrimination rather than maximizing sparsity. Metric definitions and the full per class-count tables are in the Supplementary material S3. This demonstrates that the spike-based learning rule effectively builds sparse representations, thereby reducing the energy and resource demands of the network in a hardware implementation.

**Figure 5:**
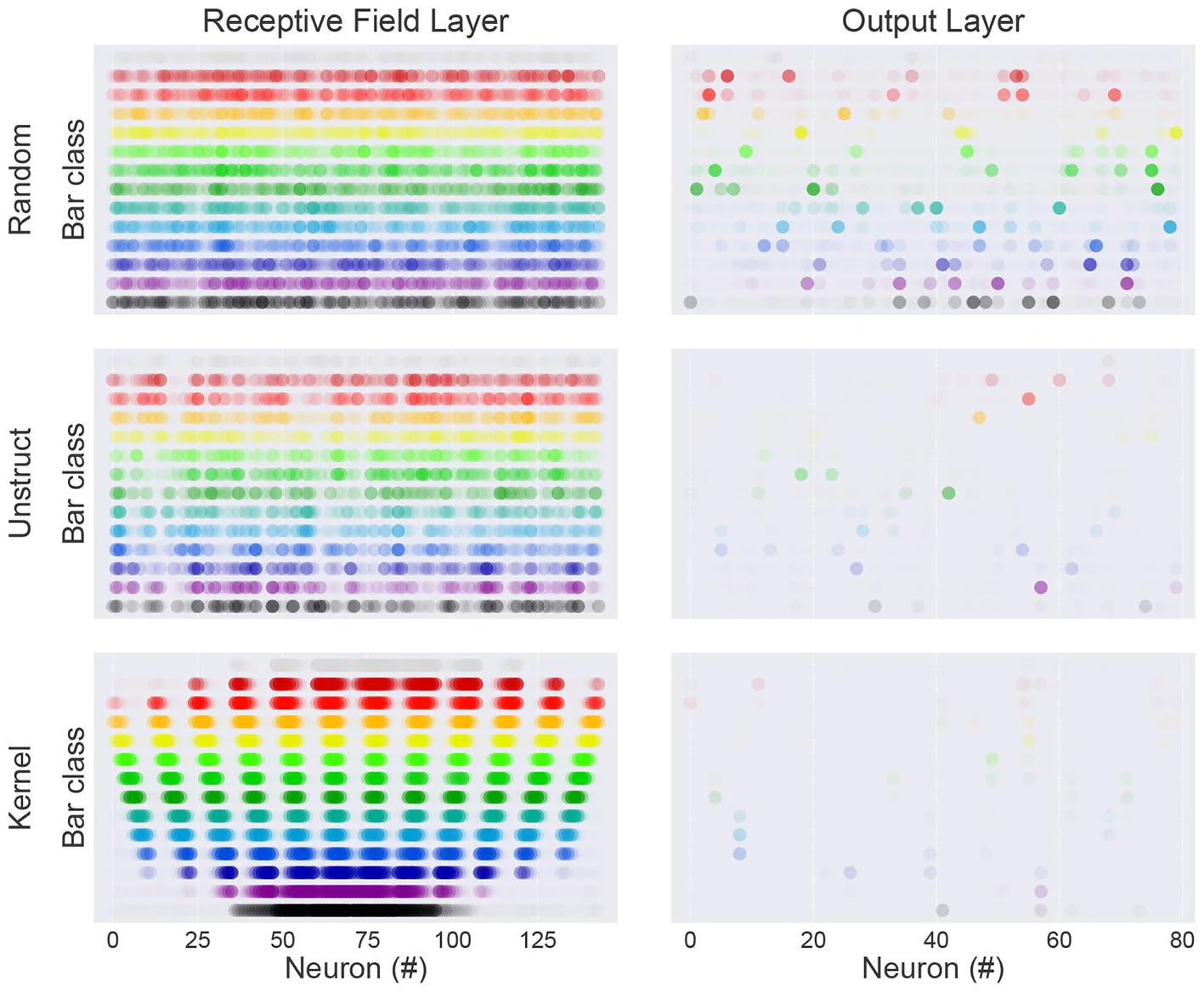
Sparsity of the network built with each receptive field condition. The color intensity of the figure represents the average activation in spikes per second of each neuron in the network per layer. All figures have been normalized for visual comparison. Every network condition has been trained to recognize bar orientations separated by 25.7° with one unit of offset. Left column, average activation of every neuron in the receptive field layer per class (y axis) and per neuron (x axis). Right column, average activation of all samples per class after training.

**Table 2:** Sparsity of the learned code for each receptive field condition, measured on the held-out test set at 14 classes and one unit of offset.

Condition	Layer	Spikes/neuron	Active frac.	Gini	Treves–Rolls *S*	Compression
Random	Receptive field	19.89	0.96	0.42	0.37	6.2 ×
	Output	5.74	0.74	0.79	0.87	
Unstructured	Receptive field	13.18	0.76	0.60	0.57	23.4 ×
	Output	1.05	0.15	0.91	0.95	
Kernel	Receptive field	35.01	0.79	0.63	0.59	148.2 ×
	Output	0.43	0.02	0.99	0.99	

Values are means over 15 seeds. Spikes per neuron and the active fraction report the raw activity of each layer. The Gini coefficient and the Treves–Rolls sparseness *S* are computed per stimulus and then averaged, following Supplementary material S3, since the aggregate Gini can read inversely to quality on a class-balanced test set. Higher Gini and higher *S* indicate a sparser code, and both give the same ordering with the output layer far sparser than the receptive field layer. Compression is the ratio of spikes emitted by the receptive field layer to those emitted by the output layer per stimulus. The output activity is compressed several fold in every condition, from 6.2 × for random to 148 × for kernel.

### 3.4 Incremental learning

This work aims at setting the basis for the development of artificial systems able to support real-world applications thanks to their scalability, flexibility, and real-time adaptability. Learning edge orientations is the perfect scenario in which an increased resolution requirement can push the system's limits. Therefore, we sought to gain a deeper understanding of how the network and learning rule would behave in response to a change in the number of classes after the system has been deployed. This setup simulated the continual learning condition of a real-world application in which not all classes may be initially presented or needed in early stages. In this case, we can initially train the system, deactivate learning for production, and reactivate learning for the same system if the specifications change. Left panel of Figure 6 represents the classification performance for all the receptive field conditions with 15 random initializations. We trained the previously shown architecture and spike-based learning rule to recognize orientations separated by 30° across all receptive field conditions for the same initial data point shown in Figure 6. After that, we retrained the same network to become sensitive to smaller changes in orientation up to 20°. For each data point in the figure, we stopped the training and calculate the testing accuracy. This procedure is shown in the figure under the condition name 'incremental learning'. For comparison purposes, we trained three different networks to discriminate orientations at 30°, 26°, and 20°, represented as the independent condition. The right panel of Figure 6 shows the retention during the incremental learning task. Each point represents the network at a different training stage, and its position on the *x* axis gives the number of classes being evaluated. These results show that the earlier classes are still retained by the network, with little or no forgetting. In comparison with independent learning, the incremental learning task shows better accuracy. The results demonstrate how the learning rule enables the network to adapt to incremental orientation detection without requiring retraining of the network from scratch. This allows our system to be incremental with the possibility of improving discrimination properties in the same hardware deployment cycle. Additionally, it would present a higher performance than training the system and deploying the network in hardware independently.

**Figure 6:**
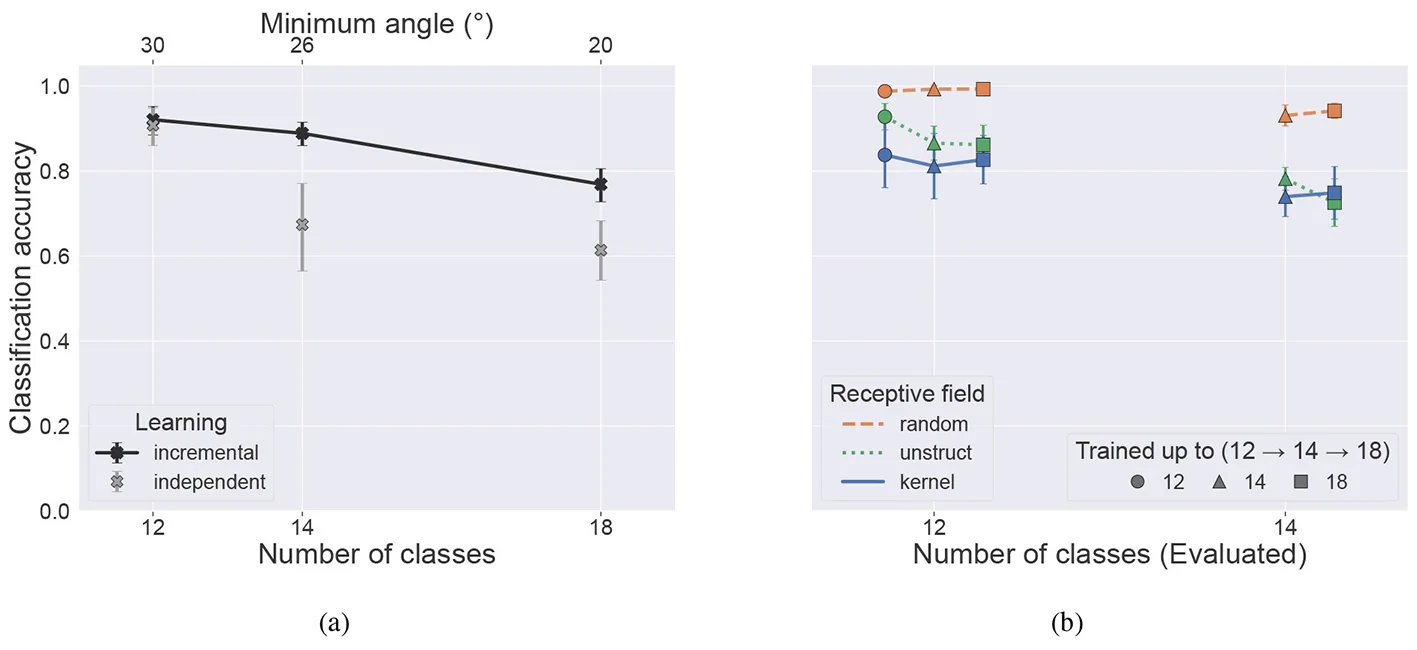
Classification accuracy for incremental vs. independent learning. We trained the orientation classification network for 12, 14 and 18 angle classes for a minimum orientation angle corresponding to 30°, 26° and 20°, presenting each dataset sequentially for the incremental condition. **(a)** Classification accuracy mean and standard deviation for the three receptive field types, trained after fifteen random initializations. We compare the results in the figure with the accuracy performance for training each of the networks with each of the datasets independently. Under all conditions, incremental learning shows a significantly higher performance in accuracy compared to independent learning. **(b)** Retention during the incremental training condition. Horizontal axis shows the evaluated set of classes, accuracy is re-measured as training continues to more classes as defined in the incremental condition (12 up to 18, legend). Marker shape gives the training stage, so each line's slope reports the retention across training per receptive field. Flat or rising means the earlier classes are kept, falling means partial forgetting. Earlier classes are largely retained, with only a mild decrease for the unstructured field at later stages.

## 4 Discussion

We developed a hardware-compatible tactile processing system that leverages bioinspired, spike-based learning with local traces and self-regulating network dynamics to efficiently extract features for orientation classification in an unsupervised fashion that supports continual, incremental learning. Inspired by biological receptive fields, the model achieves high accuracy in edge orientation discrimination and adapts naturally to increasing the orientation angular resolution. The system generates sparse, efficient codes by directly constructing receptive fields at the sensory interface, resulting in significant information compression that leads to a reduction in signal transmission.

We design receptive fields to extract relevant features for the task, drawing inspiration from biological systems. Defining a layer of predefined weights based on biological insights helps building compact models without the need for training. Edge orientation is a biological capability allocated in primary somatosensory areas. This means that the shape of the receptive fields across skin and how they convey information from the mechanoreceptors in the skin through afferent nerves play a key role in orientation tuning (Pruszynski and Johansson, 2014). We aim to leverage these insights to develop more effective artificial systems. Selecting the receptive field structure based on the nature of the data and the task to perform improves sparsity and encoded information. Additionally, hardware implementations can utilize predefined weights to shape edge-computing systems with static connections near sensors. The optimized networks presented in this manuscript demonstrate a high performance capability in discriminating orientations. The highest performance has been achieved in networks based on random receptive fields, with the second-best result in unstructured receptive field inspired by biological experiments in the literature. The networks based on kernel receptive fields, typically used to build artificial systems, have shown lower performance in comparisons across all conditions. They exhibit lower sensitivity to offsets, while still offering lower classification accuracy. Edge orientation is a relevant property in object manipulation and studies showed how humans tend to reproduce the placement of the tools and decrease the variability in the position of the grasped objects for manipulation (Scholz and Zatsiorsky, 2013; Yao et al., 2021). Our results sit alongside two closely related tactile edge orientation studies. Macdonald and colleagues classify orientation from real event-based data recorded with a NeuroTac fingertip and report a mean orientation error of 4.44 and 5.69 degrees over 18 orientations (Macdonald et al., 2022), while Dabbous and colleagues use real iCub capacitive skin and reach a minimum detectable angle near 5 degrees (Dabbous et al., 2021). Our input is synthetic, so these numbers are not directly comparable. Even so, our random receptive field reaches a comparable mean orientation error of 5.15 degrees at 18 classes, and it does so with a fully unsupervised system that learns and decides based on the network activity, replacing the external classifier read out of the other studies. Examining our findings on the difference between receptive field conditions, we can argue that the selection of the receptive field layer plays a crucial role in the performance of the system. We investigate how unstructured and random receptive fields are more effective for edge orientation classification than the receptive fields typically used in neural networks. The random field samples more distant and less correlated taxels, which decorrelates the inputs to the output layer and slightly increases the separation between classes. The unstructured field trades a little of that separation for spatially contiguous, damage-resilient clusters that match the biology. Unlike the results that yield higher values in the random receptive field, neurophysiological studies show that the unstructured condition resembles the shape of the receptive fields observed in the primate hand (Wessberg et al., 2003; Pruszynski et al., 2018). The unstructured receptive field generation may be the biological solution to randomize input selectivity while engaging as many distant mechanoreceptors as possible. Additionally, creating receptive fields with spatially contiguous taxels increases redundancy and resilience to damage. In other words, there are biological limitations on how far to connect the mechanoreceptors in a highly distributed space, which is limited by the wiring topology of the connectivity between mechanoreceptor afferents in the hand (Johansson and Åke B. Vallbo, 1979). Additional insights about the need for this sensory code compression occur at the somatosensory cortex level. The densely connected representations in primary sensory cortex become progressively compressed as information propagates toward higher cognitive areas along the sensory pathway. This is a common feature across sensory modalities in the brain and motivates topological constraints in the neural code from the periphery to higher-order cortical levels (Collins et al., 2010).

We implemented a calcium-based local learning rule, which is biologically plausible and compatible with hardware implementations, presenting an efficient solution for learning tactile features from data. We employ an unsupervised learning method based on the network's connectivity, which requires a balance between self-excitation and lateral inhibition within the network architecture. Although the learning is not supervised, the success of the task depends on the hyperparameter tuning. We tuned the hyperparameters for each network individually based on its performance in the final task. We found that the initialization of the network plays a crucial role in determining its final performance. The output number of neurons should be large to represent all input classes and account for the labeling and population vote.

Incremental learning yielded better results than independent (batch based) learning for the proposed system while showing little or no forgetting, likely due to the task's inherent structure, as new classes often overlap with those already trained. Such behavior is advantageous for lifelong learning applications that require continual adaptation, as in the proposed system. The proposed network is both shallow and energy-efficient. We can design analog circuits to encode the signal into events and fix the receptive field. The learning rule is a local, hardware-compatible rule where sensor events are directly fitted, and synapses are adjusted in task time. The network does not require offline learning, and the hyperparameter tuning is adjusted to the expected activation stimuli. These factors are adjusted in relation to the parameters in the hardware implementation, such as the tactile surface and the encoding mechanism. The reason is that a higher activity in the network than expected by the tuned hyperparameters may disrupt the balance between excitation and inhibition, and the local traces would not be well adjusted to potentiate and depress synapses. However, the hardware is immutable in the system, and once selected and tuned, the task can evolve in the number of orientations as needed. Nevertheless, additional homeostatic mechanisms could be implemented to tune the network to different levels of activity in the network.

The network exhibits sparse neural activity. Average activation in the receptive field layer is significantly higher than in the output layer, indicating efficient feature compression. Sparse activity not only improves computational efficiency but also reduces energy consumption, a key advantage for neuromorphic hardware and edge applications. This property parallels biological sensory processing, where sparse coding is thought to enhance efficiency and selectivity. In biological systems, sparse representations reduce redundancy while preserving stimulus features, enabling efficient information transmission under metabolic constraints (Attwell and Laughlin, 2001). Similarly, in artificial neuromorphic systems, sparse activity of the network has been widely used as a proxy for energy expenditure. Fewer active neurons and synaptic events translate into lower computational and communication costs (Merolla et al., 2014; Davies et al., 2018). We report a first-order, platform-agnostic estimate of the energy to classify one stimulus in Supplementary material S4. The energy consumption of the network per inference ranges between 0.16–17.18 μ*J* depending on the neuromorphic platform and classification task. While the estimate shows promising results, it has limited value since it is an estimate from spike statistics, not a hardware measurement.

This work shows how biological sensing insights can guide the design of practical artificial sensing systems. We combined bioinspired receptive fields generated with local spike-driven learning rules and self-regulating dynamics to build hardware compatible solutions that extract relevant features without supervision. We demonstrate the relevance of the design of the receptive field structure to increase performance in discrimination tasks. In the artificial tactile task, we compared biologically inspired receptive fields with a kernel based configuration designed to maximize spatially distributed information. Given the strong spatial component of the task, we expected the kernel configuration to outperform, as its structured coverage should enable an effective combination of spatial features for discrimination. However, the results show that the distributed, biologically inspired receptive fields achieve superior performance in this task. We also demonstrated how to build hardware-compatible solutions for online learning algorithms that produce naturally sparse, compressed representations for energy-efficient neuromorphic implementations. Previous work highlighted the importance of receptive field design (Dabbous et al., 2021). Our system validates this hypothesis while providing an unsupervised learning mechanism that evaluates the feature-extraction properties of the receptive-field design at the sensing layer within a biologically grounded framework. In terms of system implementations for electronic skin patches, Rahiminejad et al. (2021) found that bioinspired sensing and encoding are feasible with hardware implementations on edge devices. Our work advances these findings into an end-to-end system that links feature encoding, learning, and task performance, enabling self-calibration. We present an integrated system not only compatible with hardware implementations but also capable of self-calibrating and generating compressed representations of its sensory input space by interacting with the environment. Together, our results highlight a pathway to build adaptive, scalable, and hardware-ready touch perception systems grounded in neurobiological principles.

Several directions follow from this work. A first question is the capacity and scalability of the network and the maximum number of orientations it can resolve. A natural next step is to test recorded and dynamic tactile data with temporal structure, which would move the evaluation beyond the synthetic and instantaneous stimulus used here. Adding homeostatic mechanisms would let the network self-tune to different activity levels and ease its current dependence on per-condition hyperparameter tuning. Deploying the system on neuromorphic hardware would validate the operation-count estimate, which we report here only as a first order figure.

## Data Availability

The raw data supporting the conclusions of this article will be made available by the authors, without undue reservation.
