# Supplementary material for: Orientation detection for self-calibrating neuromorphic systems
Source: Front Comput Neurosci. 2026 Oct 23;20:1878557. doi: 10.3389/fncom.2026.1878557 (PMC13739773; doi:10.3389/fncom.2026.1878557)
Supplement: Supplementary file 1 [file Supplementary_file_1.pdf]

Supplementary Material

Orientation detection for self-calibrating neuromorphic systems

Alejandro Pequeño-Zurro[1,2,*], Chiara Bartolozzi[3], Elisabetta Chicca[1,2]

[1]Bio-Inspired Circuits and Systems (BICS) Lab, Zernike Institute for Advanced Materials, University of Groningen, Groningen, Netherlands

[2]Groningen Cognitive Systems and Materials Center (CogniGron), University of Groningen, Groningen, Netherlands

[3]Event-Driven Perception for Robotics, Istituto Italiano di Tecnologia (IIT), Genova, Italy

[*]Correspondence: `a.pequeno.zurro@rug.nl`

S1 Experimental design

All analyses use the output of the unsupervised BAR orientation network described in the main text. Network description, 225 Poisson input units → 144 fixed receptive-field (RF) units denoted in manuscript as "L0" → 80 plastic output units denoted in manuscript as "L1". Receptive fields were algorithmically generated. Output neurons were trained with the Spike-Driven Synaptic Plasticity rule under self-excitation and lateral inhibition in the output layer.

- **Conditions.** Three RF constructions (`random`, `unstructured`, `kernel`) × six class counts (8, 12, 14, 18, 20, 24 orientations).
- **Dataset**. Each stimulus is an oriented bar pressed on the simulated taxel surface and its class is represented by its orientation in the grid. The presentation lasts 400 ms per input bar in the dataset. To probe robustness we also vary the bar position with a spatial offset of 1 to 7 taxels in the x and y directions. This offset represents the placement inaccuracy of a real contact, where the same bar rarely lands on exactly the same taxel twice.
- **Seeds.** 15 seeds per configuration for statistical testing of the results. Every run uses a randomly generated recorded seed which affects the random initialization of the network.
- **Split.** Fixed 80/20 train and test split, 50 samples per class. Reported classification accuracy are on the held-out 20% test set.
- **Training.** 3 epochs, simulation 400 time steps ($dt = 1$ ms) per stimulus as the presentation of each individual stimulus last. Testing was calculated freezing the network state after training.
- **Accuracy.** Output-layer unsupervised population-vote accuracy (Section 2.5 of the main text).
- **Per-neuron data.** For the sparsity and orientation-error analyses, the per stimulus and per neuron test spike count matrices were saved for both layers after training.
- **Software.** Python 3.10, NumPy 1.26.4, SciPy 1.14.0 [1].

S2 Statistical significance

(A) Pooled. Treating the 15 accuracy values per condition as independent samples. Pairwise comparisons use the two-sided Mann–Whitney U test [2] on each of the three condition pairs.

Multiple comparisons across the three pairs, per class count, are corrected with the Holm–Bonferroni step-down procedure [3]. The effect size is the rank-biserial correlation, $r = 1 - 2U/(n_1 n_2)$ [4]. $r \to \pm 1$ means one condition almost always exceeds the other, and the sign indicates direction.

(B) Paired. Seeds are shared across conditions and matched. The Friedman test [5] is the omnibus across the three conditions at each class count. Post-hoc pairwise Wilcoxon signed-rank tests [6] are Holm-corrected across the three pairs.

The Holm procedure orders the m raw p-values ascending, $p_{(1)} \le \cdots \le p_{(m)}$, and the adjusted value of the k-th is $\max_{j \le k}(m - j + 1)\, p_{(j)}$, capped at 1.

Table S2. Pairwise significance. MWU = pooled 15-seed Mann–Whitney U (Holm-adjusted p, rank-biserial r). Wilcoxon = paired Wilcoxon signed-rank on the aligned revision seeds (Holm-adjusted p). Markers: *** $p < 0.001$, ** $p < 0.01$, * $p < 0.05$, ns = not significant.

Classes	comparison	MWU Holm p	r	MWU	Wilcoxon Holm p	Wilcoxon
8	random vs kernel	0.00015	−0.85	***	0.0045	**
8	random vs unstruct	0.031	−0.43	*	0.033	*
8	unstruct vs kernel	0.0085	−0.61	**	0.0071	**
12	random vs kernel	1.2e-05	−0.99	***	0.0059	**
12	random vs unstruct	0.00012	−0.86	***	0.0059	**
12	unstruct vs kernel	0.0041	−0.62	**	0.0098	**
14	random vs kernel	8.9e-06	−1.00	***	0.0059	**
14	random vs unstruct	8.9e-06	−0.99	***	0.0059	**
14	unstruct vs kernel	8.9e-06	−1.00	***	0.0059	**
18	random vs kernel	9.9e-06	−1.00	***	0.0059	**
18	random vs unstruct	0.0045	−0.66	**	0.039	*
18	unstruct vs kernel	0.026	−0.48	*	0.56	ns
20	random vs kernel	6.8e-05	−0.89	***	0.0052	**
20	random vs unstruct	0.0039	−0.62	**	0.041	*
20	unstruct vs kernel	9.7e-06	−1.00	***	0.00018	***
24	random vs kernel	0.0017	−0.72	**	0.035	*
24	random vs unstruct	0.0017	−0.72	**	0.076	ns
24	unstruct vs kernel	0.41	−0.16	ns	0.12	ns

Friedman omnibus per class count (aligned seeds): 8-class $\chi^2 = 17.0$, $p = 2 \times 10^{-4}$; 12-class $\chi^2 = 18.2$, $p = 1 \times 10^{-4}$; 14-class $\chi^2 = 20.0$, $p = 5 \times 10^{-5}$; 18-class $\chi^2 = 11.4$, $p = 3 \times 10^{-3}$; 20-class $\chi^2 = 21.6$, $p = 2 \times 10^{-5}$; 24-class $\chi^2 = 11.8$, $p = 3 \times 10^{-3}$.

S3 Sparsity metrics

Let a layer have N neurons and let r_i be the spike count of neuron i.

- **Gini coefficient** of a non-negative vector, from the sorted values $r_{(1)} \le \cdots \le r_{(N)}$: $G = \big(2\sum_i i\, r_{(i)}\big)/\big(N \sum_i r_{(i)}\big) - (N+1)/N$. $G = 0$ when all neurons are equal, $G \to 1$ when one neuron holds all the activity [7]. The Gini coefficient measures how unevenly the activity is distributed across neurons, running from 0 when every neuron fires equally to 1 when a single neuron carries all the activity.

- **Treves–Rolls population sparseness** for one stimulus with response r: $a = \big(\sum_i r_i\big)^2/\big(N \sum_i r_i^2\big)$, $S = 1 - a$. Here $a \in (1/N, 1]$, $a = 1$ means all neurons respond equally (dense), $a \to 1/N$ means one neuron responds (maximally sparse), and larger S is sparser [8]. The Treves and Rolls population sparseness measures how few neurons respond to a single stimulus, with higher values meaning the response is concentrated on a small subset of the population.

- **Fraction active**: fraction of neurons with $r_i > 0$.

Table S3a. Per-stimulus population sparsity of the learned output layer (L1), averaged over test stimuli.

Classes	metric	random	unstruct	kernel
8	Gini	0.274 ± 0.021	0.903 ± 0.008	0.927 ± 0.011
8	Treves–Rolls	0.242 ± 0.028	0.937 ± 0.007	0.968 ± 0.005
12	Gini	0.816 ± 0.015	0.952 ± 0.007	0.771 ± 0.008
12	Treves–Rolls	0.846 ± 0.012	0.962 ± 0.007	0.761 ± 0.008
14	Gini	0.785 ± 0.008	0.942 ± 0.005	0.986 ± 0.001
14	Treves–Rolls	0.867 ± 0.005	0.960 ± 0.004	0.986 ± 0.000
18	Gini	0.965 ± 0.002	0.447 ± 0.007	0.914 ± 0.004
18	Treves–Rolls	0.967 ± 0.002	0.383 ± 0.008	0.916 ± 0.004
20	Gini	0.958 ± 0.001	0.978 ± 0.003	0.715 ± 0.009
20	Treves–Rolls	0.963 ± 0.001	0.984 ± 0.001	0.691 ± 0.011
24	Gini	0.909 ± 0.002	0.841 ± 0.004	0.966 ± 0.003
24	Treves–Rolls	0.938 ± 0.002	0.845 ± 0.002	0.985 ± 0.000

Table S3b. Per-stimulus Treves–Rolls sparseness and mean spikes per neuron at fixed RF layer (L0) versus learned output (L1).

Classes	rf	TR L0	TR L1	spk/neuron L0	spk/neuron L1
8	random	0.424	0.242	13.74	15.88
8	unstruct	0.458	0.937	25.85	1.48
8	kernel	0.645	0.968	15.09	2.12
12	random	0.557	0.846	6.49	2.80
12	unstruct	0.545	0.962	15.28	1.24
12	kernel	0.609	0.761	26.28	15.23
14	random	0.363	0.867	20.00	5.77
14	unstruct	0.578	0.960	12.88	1.21
14	kernel	0.586	0.986	35.19	0.43
18	random	0.409	0.967	15.54	0.24
18	unstruct	0.349	0.383	56.29	66.19
18	kernel	0.506	0.916	59.21	7.52
20	random	0.510	0.963	8.52	0.77
20	unstruct	0.531	0.984	17.08	1.95
20	kernel	0.578	0.691	38.87	26.49
24	random	0.481	0.938	9.61	1.63
24	unstruct	0.352	0.845	55.10	17.91
24	kernel	0.602	0.985	28.87	2.15

S4 Energy estimate

In this section we additionally quantify the cost of running the network in compatible hardware architectures. The estimate counts only the two feed-forward synaptic projections. It excludes the lateral-inhibition and self-excitation recurrent operations in the output layer, neuronal state updates, spike generation, input encoding, memory access, on-chip routing, and static or leakage power, which on subthreshold analog hardware can be significant. The RF to output projection is assumed fully connected, a sparse deployment would lower this term. The per-SynOp energy is treated as a single constant. The figure is therefore a first-order dynamic estimate and a measurement on the target chip remains future work.

S4.1 Synaptic-operation model

On event-driven neuromorphic hardware the dominant dynamic cost is the synaptic operation (SynOp), one presynaptic spike delivered across one synapse. The number of SynOps to process one stimulus is

$$\text{SynOps} = \sum_{\text{layers}} (\text{presynaptic spikes}) \times (\text{fan-out}) = \sum_{\text{synapses}} (\text{spikes carried by that synapse}).$$

We count the two feed-forward projections, $\text{SynOps} \approx [\,\text{input spikes} \times (\text{RF synapses}/N_{\text{input}})\,] + [\,\text{RF spikes} \times N_{\text{output}}\,]$. The input to RF projection is sparse, each of the 144 RF neurons has 16 active input weights, so $144 \times 16 = 2304$ synapses, a fan-out of $2304/225 = 10.24$. The RF to output projection is treated as dense, $144 \times 80 = 11\,520$ synapses, fan-out 80, and this second term dominates.

S4.2 Per-SynOp energy

The SynOp count is multiplied by a per-SynOp energy. We use a bracket of 1–25 pJ/SynOp spanning published values for CMOS and mixed-signal neuromorphic platforms. Digital many-core designs report the upper end, Intel Loihi ≈ 23.6 pJ per synaptic operation [9], IBM TrueNorth ≈ 26 pJ per synaptic event [10]. Mixed-signal and subthreshold designs of the kind this paper targets, BrainScaleS-2 [11] and DYNAP-SE2 [12], are reported at the low-pJ end, hence the 1 pJ lower bound. The order-of-magnitude conclusion is robust across this whole bracket.

Table S4. Estimated synaptic operations and energy per classification.

Classes	rf	SynOps in→RF	SynOps RF→out	SynOps total	Energy μJ (1–25 pJ)
8	random	5,192	158,329	163,521	0.16 – 4.09
8	unstruct	5,192	297,814	303,006	0.30 – 7.58
8	kernel	5,192	173,885	179,078	0.18 – 4.48
12	random	5,066	74,262	79,328	0.08 – 1.98
12	unstruct	5,066	161,451	166,517	0.17 – 4.16
12	kernel	5,066	302,776	307,842	0.31 – 7.70
14	random	5,148	230,103	235,250	0.24 – 5.88
14	unstruct	5,148	151,271	156,419	0.16 – 3.91
14	kernel	5,148	405,376	410,524	0.41 – 10.26
18	random	5,088	178,478	183,566	0.18 – 4.59
18	unstruct	5,088	645,865	650,953	0.65 – 16.27
18	kernel	5,088	682,060	687,148	0.69 – 17.18
20	random	5,120	98,199	103,319	0.10 – 2.58
20	unstruct	5,120	196,746	201,866	0.20 – 5.05
20	kernel	5,120	447,810	452,930	0.45 – 11.32
24	random	5,076	110,298	115,373	0.12 – 2.88
24	unstruct	5,076	629,899	634,975	0.63 – 15.87
24	kernel	5,076	332,631	337,706	0.34 – 8.44

S5 Dataset

Stimuli are synthetic bars pressed on a simulated 15×15 (225-taxel) surface, then Poisson rate-encoded. Each class is one bar orientation, orientations are evenly spaced over the full circle, $\theta_k = k \cdot (360/n)$ for $k = 0 \dots n-1$, so the minimum resolvable angle is $360/n$. Contact is instantaneous, no temporal dynamics. Bars are width 2 by length 15 taxels. Encoding uses 400 time steps ($dt = 1$ ms, stimulus 350 ms plus 50 ms offset), background rate $f_{\min} = 1$ Hz, active rate $f_{\max} = 50$ Hz. Reported runs for the performance analyses use bar offset 1 taxel, 50 samples per class, split 80/20 (40 train, 10 test per class), stratified.

The encoding is stochastic. Each taxel pressure sets a mean firing rate between the background and active rates of 1 and 50 Hz, and the spike train delivered to the network is a random realisation

of an inhomogeneous Poisson process at 1 ms resolution, so at every time step each taxel emits a spike with a probability set by its rate. Same pressure pattern give different spike rasters with the same mean rate, mimicking the trial-to-trial variability of biological afferents. The 50 samples per class combine this encoding variability with the random spatial offset and small angle jitter of each presented bar, so the within-class variability arises from both the stimulus placement and the encoding noise. These spike trains are drawn once during dataset generation and stored, so they are fixed across the training runs.

To probe robustness we also vary the bar position with a spatial offset of 1 to 7 taxels in the x and y directions. This offset represents the placement inaccuracy of a real contact, where the same bar rarely lands on exactly the same taxels twice, so the offset exploration simulates the positional variability expected of a real-world deployment rather than an idealised centred presentation. The offset sweep from the result section is reported at 14 classes.

Table S5. Dataset composition per class count.

Classes n	Min angle $360/n$	Samples/class	Total	Train / Test
8	45.0°	50	400	320 / 80
12	30.0°	50	600	480 / 120
14	25.7°	50	700	560 / 140
18	20.0°	50	900	720 / 180
20	18.0°	50	1000	800 / 200
24	15.0°	50	1200	960 / 240

S6 Network architecture and receptive-field construction

Three feed-forward layers, 225 Poisson input units, 144 fixed-weight RF units (L0, LIF), and 80 plastic output units (L1, LIF) with lateral inhibition and self-excitation. $dt = 1$ ms, population_k = 3. The RF projection (225 → 144) is binary (weight `l0_w_exc` where active, else 0) and fixed. Each RF neuron has exactly 16 active input weights in all three constructions, so they differ only in the spatial arrangement of those 16 taxels.

- **kernel** (`kernels_overlap`, size$_i$ = size$_j$ = 4, overlap 3): one contiguous 4×4 taxel block per RF neuron (16 taxels), tiled across the 15×15 grid with stride 1, giving $12 \times 12 = 144$ heavily overlapping square fields.

- **unstructured** (fields 5, size_k 3, spare 1, overlap 12): 5 small contiguous sub-clusters per neuron. Each sub-cluster seeds one taxel, the first from a guaranteed pool that tiles all 225 inputs so every taxel is covered, the rest from a shuffled pool containing each input 12 times, then grows to 3 taxels by activating up to two 8-connected neighbours of the seed.

- **random** (samples 16, overlap 12): 16 taxels drawn per neuron without replacement from a pool containing each of the 225 inputs 12 times, no spatial constraint.

S7 Parameters and hyperparameter optimization

Each of the configurations per class and receptive field conditions was tuned independently to guarantee fairness with Optuna [13] using the TPE sampler, direction maximise, objective the held-out population-vote accuracy, and median pruning of unpromising trials. Each study ran under a fixed 24 h wall-clock budget averaging 400 completed trials, the fixed trial count varies across sets of parameters based on pruning conditions that stop trials after epochs when performance is low.

Table S6a. Fixed parameters.

Parameter	Value
nb_inputs / nb_hidden / nb_outputs	225 / 144 / 80
dt (ms)	1
population_k	3
epochs	3
samples per class	50
stim_length (ms)	350
offset_sample	50
min_dim / max_dim (bar $w \times L$)	(2, 15)
$f_{\min}$ / $f_{\max}$ (Hz)	1 / 50
l0_tau_epsp	6
tau_w	4000
l1_j_eff	0.03
l1_theta_dep_low / high	3 / 8
thr_w / thr_v	0.6 / 0.6
w_min / w_max	0.0 / 1.0
alpha / beta / a / b	0.1 / 0.1 / 0.1 / 0.1

Table S6b. Tuned parameters and uniform search ranges.

Parameter	Search range
`l0_w_exc`	$[0.1, 0.5]$
`l1_c_eff`	$[0.3, 0.8]$
`l1_w_inh`	$[0.01, 0.5]$
`l1_theta_pot_low`	$[1, 6]$
`l1_theta_pot_high`	$[6, 14]$
`tau_calcium`	$[10, 60]$
`l1_tau_ipsp`	$[4, 12]$
`l1_tau_epsp`	$[4, 12]$
`l1_w_exc`	$[0.1, 0.5]$
`l1_tau_excpsp`	$[3, 12]$
`l1_synaptic_percent`	$[0.02, 0.4]$

S7.1 Optimized parameter values per condition

Values used for the reported results. They were verified against the best trial of each Optuna study, identical for all conditions, differing only for three easy conditions where many trials tie at perfect accuracy so the recorded set is one of several equally optimal trials. Columns use the short names `l0we`=`l0_w_exc`, `we`=`l1_w_exc`, `wi`=`l1_w_inh`, τ_{ep}=`l1_tau_epsp`, τ_{ip}=`l1_tau_ipsp`, τ_{ex}=`l1_tau_excpsp`, τ_{ca}=`tau_calcium`, c=`l1_c_eff`, θ_{lo}=`l1_theta_pot_low`, θ_{hi}=`l1_theta_pot_high`, sp=`l1_synaptic_percent`. Times in ms.

Table S7. Optimized values, `random`.

n	l0we	we	wi	τ_{ep}	τ_{ip}	τ_{ex}	τ_{ca}	c	θ_{lo}	θ_{hi}	sp
8	0.150	0.171	0.0913	10.9	4.11	5.97	18.8	0.462	2.25	8.02	0.334
12	0.108	0.101	0.141	9.61	4.37	4.42	17.9	0.651	1.45	11.2	0.381
14	0.185	0.161	0.0505	9.47	10.8	3.32	16.8	0.480	3.13	12.0	0.283
18	0.162	0.174	0.432	11.4	8.55	3.38	34.0	0.553	1.53	12.6	0.0405
20	0.121	0.188	0.0356	11.4	8.67	3.45	54.3	0.516	3.12	13.9	0.0923
24	0.127	0.185	0.0454	5.56	10.5	3.12	12.5	0.569	1.85	13.1	0.299

Table S8. Optimized values, `unstructured`.

n	l0we	we	wi	τ_{ep}	τ_{ip}	τ_{ex}	τ_{ca}	c	θ_{lo}	θ_{hi}	sp
8	0.203	0.192	0.276	10.9	9.69	3.89	28.6	0.587	1.62	8.59	0.0733
12	0.149	0.283	0.387	11.3	8.11	3.21	15.7	0.677	2.65	9.83	0.0718
14	0.137	0.293	0.343	9.22	11.5	3.58	39.8	0.479	4.72	10.9	0.105
18	0.433	0.325	0.0282	11.7	5.95	3.29	15.4	0.391	4.20	9.16	0.372
20	0.157	0.234	0.145	9.39	5.61	4.10	27.7	0.496	3.42	6.30	0.0874
24	0.423	0.319	0.100	10.0	5.33	4.51	11.0	0.382	5.43	10.5	0.274

Table S9. Optimized values, `kernel`.

n	l0we	we	wi	τ_{ep}	τ_{ip}	τ_{ex}	τ_{ca}	c	θ_{lo}	θ_{hi}	sp
8	0.101	0.322	0.446	11.3	10.3	3.36	12.4	0.680	4.32	10.7	0.361
12	0.156	0.109	0.0365	9.56	10.1	3.13	10.0	0.601	3.46	9.45	0.400
14	0.199	0.108	0.358	7.53	9.98	3.75	22.4	0.312	4.88	14.0	0.0321
18	0.383	0.192	0.191	8.08	6.49	5.75	13.1	0.468	4.52	11.3	0.400
20	0.223	0.150	0.0223	9.48	6.57	4.00	12.5	0.382	3.37	11.6	0.204
24	0.169	0.255	0.444	7.47	10.8	3.54	20.1	0.364	4.31	6.51	0.270

S8 Orientation error

To place the network on the same axis as prior neuromorphic edge-orientation studies we report the mean orientation error in degrees, the quantity other relevant studies use, alongside the accuracy [14].

For each test stimulus the predicted class is decoded by the population vote of Section 2.5 of the main text. The orientation error is the circular distance between the predicted and the true orientation in class steps, times the angular step $360/n$, that is error $= \min(|c_{\text{pred}} - c_{\text{true}}|,\ n - |c_{\text{pred}} - c_{\text{true}}|) \times (360/n)$. Almost all errors are to an adjacent class, so the mean error is small whenever accuracy is high. Values are the mean over the test set, then the mean $\pm$ SD over seeds.

Table S10. Mean orientation error in degrees, mean $\pm$ SD over seeds.

Classes	step (°)	random	unstruct	kernel
8	45.0	0.34 ± 0.41	4.70 ± 6.73	8.07 ± 10.86
12	30.0	0.36 ± 0.41	2.33 ± 1.21	6.66 ± 3.65
14	25.7	1.05 ± 0.56	9.21 ± 5.66	20.67 ± 4.86
18	20.0	5.15 ± 0.85	57.79 ± 36.74	38.69 ± 12.56
20	18.0	5.68 ± 0.82	7.26 ± 1.03	16.51 ± 6.88
24	15.0	6.32 ± 1.05	44.10 ± 19.65	38.19 ± 14.50

References

[1] P. Virtanen, R. Gommers, T. E. Oliphant, *et al.*, "SciPy 1.0: Fundamental algorithms for scientific computing in Python," *Nature Methods*, vol. 17, pp. 261–272, 2020.

[2] H. B. Mann and D. R. Whitney, "On a test of whether one of two random variables is stochastically larger than the other," *The Annals of Mathematical Statistics*, vol. 18, no. 1, pp. 50–60, 1947.

[3] S. Holm, "A simple sequentially rejective multiple test procedure," *Scandinavian Journal of Statistics*, vol. 6, no. 2, pp. 65–70, 1979.

[4] D. S. Kerby, "The simple difference formula: An approach to teaching nonparametric correlation," *Comprehensive Psychology*, vol. 3, p. 11.IT.3.1, 2014.

[5] M. Friedman, "The use of ranks to avoid the assumption of normality implicit in the analysis of variance," *Journal of the American Statistical Association*, vol. 32, no. 200, pp. 675–701, 1937.

[6] F. Wilcoxon, "Individual comparisons by ranking methods," *Biometrics Bulletin*, vol. 1, no. 6, pp. 80–83, 1945.

[7] C. Gini, "Variabilità e mutabilità," *Studi Economico-Giuridici della R. Università di Cagliari*, vol. 3, pp. 3–159, 1912.

[8] A. Treves and E. T. Rolls, "What determines the capacity of autoassociative memories in the brain?," *Network: Computation in Neural Systems*, vol. 2, no. 4, pp. 371–397, 1991.

[9] M. Davies, N. Srinivasa, T.-H. Lin, *et al.*, "Loihi: A neuromorphic manycore processor with on-chip learning," *IEEE Micro*, vol. 38, no. 1, pp. 82–99, 2018.

[10] P. A. Merolla, J. V. Arthur, R. Alvarez-Icaza, *et al.*, "A million spiking-neuron integrated circuit with a scalable communication network and interface," *Science*, vol. 345, no. 6197, pp. 668–673, 2014.

[11] C. Pehle, S. Billaudelle, B. Cramer, J. Kaiser, K. Schreiber, Y. Stradmann, J. Weis, A. Leibfried, E. Müller, and J. Schemmel, "The brainscales-2 accelerated neuromorphic system with hybrid plasticity," *Frontiers in Neuroscience*, vol. 16, p. 795876, 2022.

[12] O. Richter, C. Wu, A. M. Whatley, G. Köstinger, C. Nielsen, N. Qiao, and G. Indiveri, "Dynap-se2: a scalable multi-core dynamic neuromorphic asynchronous spiking neural network processor," *Neuromorphic computing and engineering*, vol. 4, no. 1, p. 014003, 2024.

[13] T. Akiba, S. Sano, T. Yanase, T. Ohta, and M. Koyama, "Optuna: A next-generation hyper-parameter optimization framework," in *Proceedings of the 25th ACM SIGKDD International Conference on Knowledge Discovery & Data Mining*, pp. 2623–2631, 2019.

[14] F. L. Macdonald, N. F. Lepora, J. Conradt, and B. Ward-Cherrier, "Neuromorphic tactile edge orientation classification in an unsupervised spiking neural network," *Sensors*, vol. 22, 9 2022.
